# Utilizing a Medical Humanities-Driven Curriculum to Teach Substance Use Disorder Core Content: A Pilot Course

**DOI:** 10.1007/s40596-025-02241-4

**Published:** 2025-11-04

**Authors:** Matthew Robert Dernbach, Alaina R. Steck, Joseph E. Carpenter

**Affiliations:** Emory University School of Medicine, Atlanta, GA USA

Addiction impacts all segments of the population, with a lifetime prevalence for substance use disorder (SUD) of about 10% [1]. A sizable proportion of clinicians are uncomfortable discussing SUD with patients [2] and indeed hold negative views of patients with SUD [3], both of which can contribute to reluctance to provide addiction-related interventions [4]. Whereas all physicians at some point will participate in the care of patients with SUD, medical schools have historically devoted less time to SUD than similarly prevalent illnesses [5]. Consequently, there are calls for increased time devoted to SUD content in medical training, with an emphasis on creative coursework that incorporates education on the “attitudes and skills” involved in the treatment of patients with SUD, which will not only improve the delivery of evidence-based care but also improve the clinical culture around SUD [6].

At the same time, the Association of American Medical Colleges (AAMC) has called for medical humanities content to be included in medical education [7]. Moreover, medical students desire increased inclusion of the medical humanities into their medical education [8]. Unfortunately, there has been relatively minimal progress in medical schools doing so, in part due to the perception that medical humanities education lacks quantitative and objective rigor [9]. Nevertheless, medical humanities-driven teaching is an engaging and efficacious means to improve clinical knowledge, skill, and empathy. The medical humanities utilize interdisciplinary content and practices, with an emphasis on patient-centered and relationship-based medicine rather than technical expertise, for the purpose of the moral and professional development of medical trainees [10]. The benefit of medical humanities has been well demonstrated to include improvements in medical knowledge and ethics, clinical acumen, and empathy [11–13]. Medical humanities coursework has been successfully applied to enhance end-of-life care, professionalism, clinical communication, and clinical observation [14], and addressing stigma [15]. However, there is limited research on the development of humanities-driven coursework to teach SUD-related content to medical trainees.

To fill gaps in both SUD and medical humanities education, we developed and piloted a novel medical humanities-based SUD curriculum at Emory University School of Medicine (Emory SOM) to teach SUD-related core content. Recognizing that medical humanities education should be competency-based and “include measuring learner outcomes beyond satisfaction with the course or program and should follow sound scholarly practices [7],” in Spring 2024, we distributed a survey to all medical students at Emory SOM to identify gaps in medical school SUD curricula. The survey utilized a combination of Likert scale and multiple-choice questions, aiming to determine the extent of respondents’ perceived and objective knowledge gaps as they relate to each of the American Society of Addiction Medicine (ASAM) core content learning objectives [16]. We identified several themes from self-identified gaps in addiction knowledge, and the themes that seemed most adaptable to a medical humanities-based education included comfort discussing SUD history and diagnosis with patients, as well as comfort with assessing a patient’s readiness to change their substance use-related behavior [17].

Drawing from these findings, we designed a medical humanities-based SUD course for medical students of any training level. The purpose of the course was to improve learners’ comfort in engaging patients in a discussion about their substance use and to increase comfort in assessing patients’ readiness to change their substance use-related behavior, while also disseminating an evidence-based SUD educational resource. The course included pre-reading of medical humanities materials with associated in-class interactive discussion of the material with students and clinician facilitators. Course efficacy was evaluated with surveys of subjective comfort as applied to addiction generally and to specific clinical scenarios. The Emory University Institutional Review Board (IRB) deemed this study to be classroom activity, and therefore exempt from full IRB review and oversight.

## Curriculum Development and Implementation

Our curriculum included four 2-h individual seminars. Each seminar included two clinician facilitators: a psychiatrist and medical toxicology fellow (MRD) and an emergency medicine, addiction medicine, medical toxicology physician (either ARS or JEC). In 2024, we implemented the pilot course across two groups, one of medical student volunteers and one of medical students who took the course for elective credit. No pre-requisite SUD-related knowledge was required.

Third year medical student (MS 3) and fourth year medical student (MS 4) participants were volunteers recruited via the survey distributed to all Emory SOM medical students referenced above. Second year medical student (MS 2) participants took the course for elective credit by choosing the course from a variety of elective offerings listed in a course catalog. MS 2’s at Emory SOM are required to take an elective as part of the Foundations Phase of their curriculum. Foundations electives can have flexible contexts, with the primary requirement being that the elective is approved to be offered by the Emory SOM elective committee. In 2024, there were 37 foundations electives offered at Emory SOM, with a median MS 2 attendance of 2 and a mode attendance of 2.

The artistic materials included in the syllabus (Table 1) were chosen by the course facilitators based on their diversity of perspectives as well as their relevance to the “Top Ten Things Every Physician Should Know About Addiction,” which is an evidence-based educational resource intended for all physicians that has been endorsed by multiple medical professional societies, including the American Psychiatric Association (APA), American Academy of Addiction Psychiatry (AAAP), American Academy of Family Physicians (AAFP), and ASAM [18]. Some material was edited for length (e.g., only select chapters from books were included in the syllabus). We did not conduct a systematic search for material or a formal thematic analysis. The material was primarily discovered through independent research or recommendations from patients that occurred within the course of clinical practice.

**Table 1:** Examples of artistic works that could be included in a medical humanities-based substance use disorder (SUD) curriculum, arranged by primary SUD focus

Primary substance use disorder focus of work	Type of media	Reference
Alcohol	Parker D. Big Blonde. Penguin Classics, 2022	Fiction
	Akbar K. Calling a Wolf a Wolf. Farmington, ME: Alice James Books, 2017	Poetry (complete work)
	Williams E. Commute: An Illustrated Memoir of Female Shame. New York: Abrams Comicarts, 2019	Graphic novel
	Knapp C. Drinking: A Love Story. New York: Dell Pub., 1997	Nonfiction (autobiography)
	Prouse L. Final Approach: Northwest Airlines Flight 650, Tragedy and Triumph. CreateSpace Independent Publishing Platform, 2011	Nonfiction (autobiography)
	Zemeckis R, director. Flight. Paramount Pictures, 2012	Film
	Allen C. I’m Black and I’m Sober: The Timeless Story of a Woman’s Journey Back to Sanity. Center City, MN: Hazelden, 1995	Nonfiction (autobiography)
	Wertz J. Impossible People: A Completely Average Recovery Story. New York: Black Dog & Leventhal Publishers, 2023	Graphic novel
	Sutherland J. Last Drink to LA: Confessions of an AA Survivor. Short Books, 2014	Nonfiction (autobiography)
	Figgis M, director. Leaving Las Vegas. MGM/UA Distribution Co., 1995	Film
	Karr M. Lit: A Memoir. New York: Harper Perennial, 2010	Nonfiction (autobiography)
	Hill AB. Long Walk Out of the Woods: A Physician’s Story of Addiction, Depression, Hope, and Recovery. Central Recovery Press, 2019	Nonfiction (physician perspective)
	Erofeev V. Moscow to the End of the Line. Translated by H. William Tjalsma. Northwestern University Press, 1992	Fiction
	Doucet J. My New York Diary. Montreal: Drawn & Quarterly Publications, 1999	Graphic novel
	Bukowski C. On Drinking. Edited by Abel Debritto. Ecco, 2019	Miscellaneous
	Akbar K. Portrait of the Alcoholic. Sibling Rivalry Press, LLC., 2017	Poetry (complete work)
	Winehouse A. “Rehab.” Back to Black, Island, 2006	Music
	Ollmann J. The Abominable Mr. Seabrook. Drawn & Quarterly, 2017	Graphic novel
	Ames J. The Alcoholic. Vertigo, 2009	Graphic novel
	Walls J. The Glass Castle. New York: Scribner, 2006	Nonfiction (autobiography)
	Roth J. The Legend of the Holy Drinker. Translated by Michael Hoffman. Granta Books, 2022	Fiction
	de Saint-Exupéry A. The Little Prince. Translated by Richard Howard. Clarion Books, 2000	Fiction
	Jackson C. The Lost Weekend. Knopf Doubleday Publishing Group, 2013	Fiction
	Hiney T and MacShane F (eds). The Raymond Chandler Papers: Selected Letters and Nonfiction 1909–1959. Grove Press, 2002	Miscellaneous
	Alyan H. The Twenty-Ninth Year. Ecco, 2019	Poetry (complete work)
	The Avett Brothers. “When I Drink.” The Gleam, Ramseur Records, 2006	Music
	Albee E. Who’s Afraid of Virginia Woolf? Berkley, 2006	Play
Nonspecific substances	Jane's Addiction. “Jane Says.” Nothing’s Shocking, Warner Bros., 1988	Music
	B.o.B, featuring Priscilla. “John Doe.” Underground Luxury, Rebel Rock, Grand Hustle, Atlantic, 2013	Music
	K’s Choice. “Not an Addict.” Paradise in Me, Jet, 1995	Music
	Marder D, director. Sound of Metal. Amazon Studios, 2019	Film
	Red Hot Chili Peppers. “Under the Bridge.” Blood Sugar Sex Magik, Warner Bros., 1992	Music
Opioids	Baudelaire C. Artificial Paradises. Translated by Kirk Watson. Kensington Publishing Corp., 1998	Nonfiction (autobiography)
	U2. “Bad.” The Unforgettable Fire, Island, 1984	Music
	Felscherinow VC, Hermann K, Rieck H. Christiane F. Bantam, 1982	Nonfiction (autobiography)
	MacIndoe G. Coming Clean. 2014	Photography
	De Quincey T. Confessions of an English Opium Eater. Penguin Classics, 2003	Nonfiction (autobiography)
	Kingsolver B. Demon Copperhead. Harper, 2022	Fiction
	Strong D, creator. Dopesick. Hulu, 2021	Television
	Levinson S, creator. Euphoria. HBO, 2019	Television
	Grinspoon P. Free Refills: A Doctor Confronts His Addiction. Grand Central Publishing, 2016	Nonfiction (autobiography)
	Aron NR. Good Morning, Destroyer of Men’s Souls: A Memoir of Women, Addiction, and Love. Crown, 2020	Nonfiction (autobiography)
	Krosoczka JJ. Hey, Kiddo: A Graphic Novel. Graphix, 2018	Graphic novel
	the Velvet Underground. “Heroin.” The Velvet Underground & Nico, Verve, 1967	Music
	Smith C. Heroin and Other Poems. W. W. Norton & Company, 2000	Poetry (complete work)
	Vance JD. Hillbilly Elegy: A Memoir of a Family and Culture in Crisis. Harper, 2016	Nonfiction (autobiography)
	Wallace DF. Infinite Jest. Back Bay Books, 2006	Fiction
	Burroughs WS. Junky. Grove Press, 2012	Fiction
	Burroughs WS. Naked Lunch. Grove Press, 2013	Fiction
	Smith E. “Needle in the Hay.” Elliott Smith, Kill Rock Stars, 1995	Music
	Brixius L, Dunsky E, Wallem L, creators. Nurse Jackie. Showtime, 2009	Television
	Williams WC. The Doctor Stories. New York: New Directions, 2018	Fiction
	Vuong O. On Earth We’re Briefly Gorgeous. Penguin Press, 2019	Fiction
	Carr BA. Opioid, Indiana. Soho Press, 2019	Fiction
	Stahl J. Permanent Midnight: A Memoir. Process, 2005	Nonfiction (autobiography)
	Lyon J. Pill Head: The Secret Life of a Painkiller Addict. Grand Central Publishing, 2009	Nonfiction (autobiography)
	Schonberg J, Bourgois P. Righteous Dopefiend. University of California Press, 2009	Photography
	U2. “Running to Stand Still.” The Joshua Tree, Island, 1987	Music
	Stein M. The Addict: One Patient, One Doctor, One Year. William Morrow Paperbacks, 2010	Nonfiction (physician perspective)
	Carroll J. The Basketball Diaries. Penguin Books, 1987	Nonfiction (autobiography)
	Ditlevsen T. The Copenhagen Trilogy. Translated by Tiina Nunnally and Michael Favala Goldman. Farrar, Straus and Giroux, 2021	Nonfiction (autobiography)
	Preminger O, director. The Man with the Golden Arm. United Artists, 1955	Film
	Lee H. To Kill a Mockingbird. Harper Perennial Modern Classics, 2002	Fiction
	Young N. “The Needle and the Damage Done.” Harvest, Reprise, 1972	Music
	Schatzberg J, director. The Panic in Needle Park. 20th Century-Fox, 1971	Film
	Simon D, creator. The Wire. HBO, 2002	Television
	Boyle D. Trainspotting. PolyGram Filmed Entertainment, 1996	Film
Psychedelics	Huxley A. The Doors of Perception. Harper Perennial Modern Classics, 2009	Nonfiction (autobiography)
	Miller J and Koral R (eds). White Rabbit: A Psychedelic Reader. Chronicle Books LLC, 1995	Miscellaneous
Stimulants	Sheff D. Beautiful Boy: A Father’s Journey Through His Son’s Addiction. Mariner Books, 2009	Nonfiction (autobiography)
	Letts T. Bug. Northwestern University Press, 2006	Play
	Marnell C. How to Murder Your Life: A Memoir. Simon & Schuster, 2017	Nonfiction (autobiography)
	Davidson T (ed). Intoxication: An Anthology of Stimulant-Based Writing. Serpent’s Tail, 1998	Miscellaneous
	Clegg B. Portrait of an Addict as a Young Man: A Memoir. Back Bay Books, 2011	Nonfiction (autobiography)
	Third Eye Blind. “Semi-Charmed Life.” Third Eye Blind, Elektra, 1997	Song
	Doyle AC. The Sign of Four. Penguin Classics, 2001	Fiction
	Verghese A. The Tennis Partner. Harper Perennial, 2011	Nonfiction (physician perspective)
	Clark L. Tulsa. Grove Press, 2000	Photography
	Sheff N. Tweak: Growing Up on Methamphetamines. Atheneum Books for Young Readers, 2009	Nonfiction (autobiography)
Various specific substances	Santora PB, Dowell ML, Henningfield JE (eds). Addiction and Art. Johns Hopkins University Press, 2010	Painting
	Frey J. A Million Little Pieces. Vintage, 2005	Fiction
	Erowid. Erowid Experience Vaults. https://www.erowid.org/experiences/. Accessed 1 July 2025	Miscellaneous
	Thompson HS. Fear and Loathing in Las Vegas: A Savage Journey to the Heart of the American Dream. Vintage, 1998	Fiction
	Sparks B. Go Ask Alice. Simon & Schuster Books for Young Readers, 2006	Fiction
	Eminem. “Going Through Changes.” Recovery, Aftermath, Shady, Interscope, WEB, 2010	Song
	Morris H. Hamilton’s Pharmacopeia. Viceland, 2016	Television
	Marks H. Howard Marks’ Book of Dope Stores. Vintage, 2001	Miscellaneous
	Mettler S, creator. Intervention. A&E, 2005	Television
	Maté G. In the Realm of the Hungry Ghost. Vintage Canada, 2009	Nonfiction (physician perspective)
	Johnson D. Jesus’ Son. Picador, 2009	Fiction
	Pollock DR. Knockemstiff. Anchor, 2009	Fiction
	O'Neill E. Long Day's Journey into Night. Yale University Press, 2002	Play
	Melechi A (ed). Mindscapes: An Anthology of Drug Writings. Mono, 2002	Miscellaneous
	Steinberg N and Bader S (eds). Out of the Wreck I Rise: A Literary Companion to Recovery. University of Chicago Press, 2016	Poetry (complete works)
	Macmillan D. People, Places and Things. Oberon Books, 2016	Play
	Shulgin A, Shulgin A. PiHKAL: A Chemical Love Story. Transform Press, 1990	Miscellaneous
	Aronofsky D, director. Requiem for a Dream. Artisan Entertainment, 2000	Film
	Longyear BB. Saint Mary Blue. Steeldragon Pr, 1988	Fiction
	Maurer DD. Sobriety: A Graphic Novel. Hazelden, 2014	Graphic novel
	Cuban B. The Addicted Lawyer: Tales of the Bar, Booze, Blow, and Redemption. Post Hill Press, 2017	Nonfiction (autobiography)
	Storer C, creator. The Bear. FX on Hulu, 2022	Television
	Shulgin A, Shulgin A. TiHKAL: The Continuation. Transform Press, 2002	Miscellaneous
	Rudgley R (ed). Wildest Dreams: An Anthology of Drug-Related Literature. Thistle Publishing, 2014	Miscellaneous

Items marked by an asterisk (*) were included in the pilot course syllabus

Students pre-read the materials for each seminar, which took about 30–60 min of preparation time per seminar. The seminar discussion began with an overview of the evidence base for two to three of the items from “Top Ten Things Every Physician Should Know About Addiction,” which were related to the associated readings for the respective session. Then the students engaged in a facilitated reflection of the readings. Each 2-h seminar included discussion of between five and eight artistic works, depending on word length. The course facilitators utilized eclectic literary analysis techniques to conduct the course, such as close reading, reader-response criticism, deconstructionist criticism, and psychoanalytic criticism [11]. Text-based discussion was initially guided by broad questions to prompt reflection (Table 2), with more directed questions being based on the specifics of the work being discussed and the flow of student reflection.

**Table 2:** Example questions to guide student reflection

• What did you feel and think when you read this work? How did those change over the course of engaging with the work?
• What assumptions about addiction did the narrator seem to introduce?
• Consider the character(s) in the work as if they were your patient. What questions do you have for this character? What else would you like to know?
• How does this character describe their experiences, both while using and not using substances?
• What substance effects is this character experiencing? Which of these effects seem most/least interesting or desirable to you?
• What factors are contributing to this character’s substance use?
• How has addiction impacted this character’s life? How has the character’s addiction impacted the lives of those around them?
• How would you characterize this character’s insight into their addiction? At what stage of change are they?
• How does this character describe or define themself?
• How does this character conceptualize their addiction? How do they characterize their relationship with substances?
• What model of addiction seems to align best with this character’s experience?
• How does this character’s experience of addiction differ from the experience of another character’s that we have discussed?
• How is this character’s experience of addiction consistent/inconsistent with what we have learned about addiction from the scientific literature?

In addition to directly engaging the texts, the reflection portion also afforded students the opportunity to ask questions about SUD, to discuss particular SUD-related issues that have arisen during their medical training or other experiences, or to raise concerns that they have regarding engaging with patients with SUD in the future. Facilitators also elaborated on the textual examples by discussing the history and management of similar clinical cases that have arisen during their practice.

Students completed an anonymous pre-course survey that included Likert-scale questions to assess their subjective comfort with the learning objectives (1 = *not at all comfortable*, 10 = *completely comfortable*), their comfort engaging with 25 different SUD-related clinical scenarios (1 = *not at all comfortable*, 5 = *completely comfortable*), and their awareness of the ten individual items of the “Top Ten Things Every Physician Should Know About Addiction” (1 = *not at all aware*, 5 = *fully aware*). Students completed similar anonymous surveys immediately following the completion of the course and again 5 weeks after completion of the course. The wording of the clinical case scenarios was altered slightly between each survey administration time. Immediately following the completion of the course, feedback regarding course quality and suggestions for improvement were also requested via additional Likert-scale questions for the quality of the course and reading materials (1 = *very poor*, 10 = *outstanding*) and the degree to which the reading materials were essential to achieving the course learning objectives (1 = *not at all essential*, 10 = *completely essential*), as well as free response. Quantitative data were reported as means and standard deviations (SD).

## Outcomes

Course participants included two MS 2s, five MS 3s, and one MS 4. All participants were female. All participants completed all four seminars in their respective groups, and all participants completed each of the three surveys.

Students reported neutral initial comfort engaging patients in a conversation about their substance use prior to the course (mean = 5.0, SD = 2.2), with elevated comfort immediately following the course (mean = 7.9, SD = 0.9), which was maintained 5 weeks post-course (mean = 7.8, SD = 1.6). Similarly, students reported neutral initial comfort assessing a patient’s readiness to change their substance-use related behavior prior to the course (mean = 5.3, SD = 1.8), with elevated comfort immediately following the course (mean = 7.6, SD = 1.2), which was slightly increased 5 weeks post-course (mean = 8.0, SD = 1.3).

Students reported overall neutral initial comfort navigating various SUD-related clinical scenarios prior to the course (mean = 2.4, SD = 0.6), with elevated comfort immediately following the course (mean = 3.9, SD = 0.3), which was maintained 5 weeks post-course (mean = 3.7, SD = 0.4).

Students reported overall high initial awareness of the items for “Top Ten Things Every Physician Should Know About Addiction” (mean = 4.5, SD = 0.4), with elevated awareness immediately following the course (mean = 5.0, SD = 0.0), which was maintained 5 weeks post-course (mean = 5.0, SD = 0.0).

Students rated the overall quality of the course highly (mean = 8.4, SD = 0.9), and rated the overall quality of the reading materials highly (mean = 8.6, SD = 1.0). They also rated the reading materials as highly important to achieving the course learning objectives (mean = 8.4, SD = 1.0). When asked if they would recommend this course to other medical students, six responded “yes,” two responded “maybe,” and none responded “no.”

## Discussion and Future Directions

Medical student knowledge of SUD content was improved, the learning objectives were met, and awareness of the evidence-based resource was enhanced by the course. Moreover, student comfort and knowledge were retained over time. The initially neutral comfort of students with engaging in SUD-related patient encounters emphasizes the need for additional addiction-related content in medical education. The course also demonstrated that a medical humanities-driven curriculum can feasibly and effectively teach addiction-related core content and learning objectives.

Medical humanities materials, such as those included in the course syllabus, afford the opportunity for students to be rapidly exposed to the similarities and differences in the lived experience of individuals with a variety of SUDs, as well as the language that individuals might use to describe those experiences. Contrasting this to a strictly clinical setting, this breadth of exposure might require a longitudinal experience in order to develop the therapeutic alliance in which a patient would feel comfortable disclosing the details of their personal and interior life.

Moreover, patients with SUD may mistrust clinicians, in part due to the sensitive and stigmatized nature of their experiences [19]. In response to this, clinicians may facilitate a discussion of SUD with patients through a personalist, non-judgmental, and empathetic approach—all of which can be fostered through a medical humanities education. Indeed, clinician exposure to the experiences and narratives of individuals with SUD has been demonstrated to be a significant stigma intervention with a lasting effect [20].

This pilot course has several limitations. The small sample size and the fact that the course was implemented at a single institution limit its generalizability. Outcomes were also based on students’ subjective reports as opposed to objective assessments of skills. Self-reported comfort with written clinical scenarios may not translate to comfort with real-world clinical encounters, and future iterations may include students participating in encounters with standardized patients, peer support specialists, or actual patients. Future assessments may include a simulation or Objective Structured Clinical Examination (OSCE) at the end of the course. Future iterations of the course may also assess longer-term educational outcomes.

The course was implemented at a moderately sized medical school (at the time of the study, Emory SOM had 611 enrolled medical students across all years). Implementing the course at a smaller medical school may require some modifications in order to ensure that course enrollment affords the opportunity for varied perspectives. For instance, the medical school curriculum might require students to participate in a medical humanities elective, for which participation in this course would satisfy that requirement. Alongside medical student enrollees, course enrollment might also be opened up to any interested medical trainee or medical staff associated with the medical school. Course facilitators would ideally have experience teaching humanities and experience treating addiction in a clinical environment. Medical schools that do not have medical humanities, addiction medicine, or addiction psychiatry departments may need to develop relationships with community partners to identify facilitators with relevant teaching experience.

There is limited published data to suggest that there is a preponderance of female physicians entering the field of addiction medicine or addiction psychiatry, or that there is a preponderance of female medical students engaging in medical humanities courses. While it remains unclear why participation in our curriculum was limited to females, we suspect that factors specific to Emory SOM may have contributed. At the time this study was conducted, total medical student enrollment at Emory SOM was about 60–70% female. Also, at the time this study was conducted, medical student membership in the Emory SOM addiction medicine interest group was predominantly female, while membership in the medical humanities interest group was generally evenly split between females and males. The relatively small sample size of our curriculum may have also contributed. We anticipate that multisite applications of this curriculum would demonstrate more evenly distributed demographics among participants.

The pilot course syllabus only included written material, and did not include audiovisual media ( paintings, photography, plays, television, film, music, etc.). Future iterations of this course could revise the syllabus to include more diverse authors, experiences, and media (Table 1). It is unclear how the possible inclusion of listening/viewing sessions would impact seminar discussion, time allocation, or learning outcomes. Future course iterations may also pair alternative evidence-based resources with the artistic material. Such resources would likely feature general SUD-related themes and clinical principles as opposed to highly specific research findings. The panel of facilitators could also be expanded to include clinicians from various specialties and/or peer support specialists. Additional work could be conducted to develop a standardized education for course facilitators to be able to optimally conduct the course with consistent rigor.

Finally, the learning objectives only focused on two of the ASAM core content. Whereas some core content is more amenable than others to application to a medical humanities-based curriculum, further research is needed to determine what qualities of a core content learning objective make it a profitable target for a medical humanities-based curriculum.

## Data Availability

Not applicable.
